# Comparison of current practices of cardiopulmonary perfusion technology in Iran with American Society of Extracorporeal Technology’s standards

**DOI:** 10.15171/jcvtr.2016.14

**Published:** 2016-06-28

**Authors:** Amir Faravan, Nooredin Mohammadi, Alireza Alizadeh Ghavidel, Mohammad Zia Toutounchi, Ameneh Ghanbari, Mehran Mazloomi

**Affiliations:** ^1^Department of Critical Care Nursing, Center for Nursing Care Research, Nursing and Midwifery Faculty, Iran University of Medical Sciences, Tehran, Iran; ^2^Department of Critical Care Nursing, Nursing and Midwifery Faculty, Iran University of Medical Sciences, School of Nursing and Midwifery, Flinders University, Australia; ^3^Heart Valve Disease Research Center, Rajaie Cardiovascular Medical & Research Center, Iran University of Medical Sciences, Tehran, Iran; ^4^Rajaie Cardiovascular Medical & Research Center, Cardiac Surgery Department, Iran University of Medical Sciences, Tehran, Iran

**Keywords:** Perfusion, Standard, Cardiopulmonary bypas

## Abstract

***Introduction:*** Standards have a significant role in showing the minimum level of optimal optimum and the expected performance. Since the perfusion technology staffs play an the leading role in providing the quality services to the patients undergoing open heart surgery with cardiopulmonary bypass machine, this study aimed to assess the standards on how Iranian perfusion technology staffs evaluate and manage the patients during the cardiopulmonary bypass process and compare their practice with the recommended standards by American Society of Extracorporeal Technology.

***Methods:*** In this descriptive study, data was collected from 48 Iranian public hospitals and educational health centers through a researcher-created questionnaire. The data collection questionnaire assessed the standards which are recommended by American Society of Extracorporeal Technology.

***Results:*** Findings showed that appropriate measurements were carried out by the perfusion technology staffs to prevent the hemodilution and avoid the blood transfusion and unnecessary blood products, determine the initial dose of heparin based on one of the proposed methods, monitor the anticoagulants based on ACT measurement, and determine the additional doses of heparin during the cardiopulmonary bypass based on ACT or protamine titration. It was done only in 4.2% of hospitals and health centers.

***Conclusion:*** Current practices of cardiopulmonary perfusion technology in Iran are inappropriate based on the standards of American Society of Cardiovascular Perfusion. This represents the necessity of authorities’ attention to the validation programs and development of the caring standards on one hand and continuous assessment of using these standards on the other hand.

## Introduction

Standards have a significant role in showing the minimum optimum and the expected performance, targeting, accurate determination of hospital current status, educational programs, assessment, supervision, and direction of the organization activities. On the other hand, the clinical personnel performance has a great effect on increasing the efficiency and productivity of hospital, and patient satisfaction as well. The cardiac operating room is one of the important areas in the hospital where the observation of standards and appropriate performance of personnel leads to providing the appropriate medical services.^1^ It makes the arrangements to evaluate the performance, save the time and budget, and facilitate the decision making.^2^ In fact, the professional standards reflect the levels of competence and sufficiency at all levels of the process of using the cardiopulmonary bypass.

Indeed, the professional standards guide to the clinical measurements. The purpose of professional standards is to provide the guide to direct the perfusionists, decrease the complications and risks due to the use of cardiopulmonary bypass machine, and increase the quality of services provided to the patients. Professional standards ensure the responsibility of the professional individuals against the decisions, measurements, and their competence during their profession.^3^ The perfusion safety and reduction of the potential complications and risks in using the cardiopulmonary bypass machine is a multi-faceted issue that involves a wide range of cases. The issue includes not only the equipment used in cardiopulmonary bypass, but also the design time, machine development, perfusion methods, and the surgical technique. The process also includes issues such as perfusionists’ training and their awareness to deal with the events that may occur during the surgery at the same time in the operating room which require the proper and effective interaction between the surgeon, anesthesiologist, nurse anesthetist, and perfusionist.^4^

The importance and report of the common use of healthcare quality assessment provided in institutions and hospitals have been significantly verified.^5^ Regarding the crucial importance of observing the standards and the personnel performance in the organization, and reviewing the published studies on the observation of perfusion standards, there was no research to evaluate the situation of standards in Iran. This article was based on a large study aimed to compare the situation of perfusion in Iran with the standards of American Society of Extracorporeal Technology (AmSEXT). Therefore, the article studied the observation of standards of the patients in the cardiopulmonary bypass process in Iran and compared it with the standards of AmSECT.

## Materials and Methods

This is a descriptive-comparative study carried out in the census sampling method. The present study received the ethical approval from the ethics committee of Iran University of Medical Sciences and Health Services. The subjects of this study were from the university medical centers and public hospitals with active cardiac operating room. In order to perform the study, first, a list of university medical centers and qualified hospitals, and contact numbers and names of the authorities in each of these centers were obtained by Iranian Society of Extracorporeal Technology. Then, the questionnaire prepared according to the standards proposed by the Iranian Perfusionists Association was sent through the registered mail to 60 university medical centers and hospitals and an envelope was located within each sent envelope to return the questionnaire. It should be noted that along with the questionnaire sent to the participants, there was an informed consent form at the top of the data collection questionnaire there was an informed consent form and a description about the research purposes. Having filed out the informed consent form, the participants attempted to fill the data collection questionnaire out. The researcher tracked the return of questionnaires from the participants at regular intervals through the telephone from the participants. In the case of any queries or ambiguities by the participants about the questionnaire, participants were answered.

The data collection tool in this study was the questionnaire prepared according to the standards developed and provided by AmSECT in 2013. In addition, the reverse translation was done for using the questionnaire in the study. In this regard, standards were initially translated into fluent Persian and then, this translation was retranslated into English by an English specialist. The Persian text was corrected according to its compatibility with English text. The face validity of instruments was evaluated by 12 professors of Iran University of Medical Sciences and the necessary changes in the questionnaire were made based on their opinions. The questionnaire was developed by the standard tools, so the evaluation of reliability of instruments was unnecessary. The questionnaire included 15 standards categorized in three parts of the standards of the patients, perfusionists and perfusion protocol. It should be noted that in this article, only the standard of the patients was reported. The standards of the patients had six parts including the standards of the patient blood pressure (4 items), standards of the circulating blood flow (4 items), standards of gas exchange in patients (5 items), standards of the blood transfusion and blood products (5 items), standards of using the anticoagulants during the cardiopulmonary bypass (8items), and standards of perfusion record (9 items).

The researcher contacted the participating centers which had not fully filled out the questionnaires to complete the unanswered questions. Thus, all the questions in the returned questionnaires were completed and no unanswered question was left. The data collected from the university medical centers and public hospitals with active cardiac operating room were imported into SPSS v.13 and the results were analyzed as the descriptive statistics. Regarding the data report and analysis, the situation of the health centers in Iran in observing the standards was categorized. Therefore, observing the standards by 70% or higher was considered favorable, 50% to 70% relatively favorable and lower than 50% unfavorable.

## Results

In this study, 80% of questionnaires were returned. Table 1 shows Frequency and percentage of “standard observation”.

**Table 1:** Frequency and percentage of “standard observation”

**Standard**	**Evaluation Criteria**	**Yes**		**No**	
		**No.**	**%**	**No.**	**%**
Blood pressure	1. Do the perfusionists determine the required blood pressure in the operation by interacting with the surgeon before the onset of the cardiopulmonary bypass process?	33	68.8	15	31.2
	2. In order to maintain the blood pressure within the specified range, do the perfusionists interact with the surgical team during the cardiopulmonary bypass?	33	68.8	15	31.2
	3. Are the changes in blood pressure recorded and delivered to the surgeon?	19	39.6	29	60.4
	4. Are the measures taken to prevent the blood pressure changes recorded and delivered to the surgical team?	11	22.9	37	77.1
Blood flow rate	1. Is the blood flow rate determined before starting the cardiopulmonary bypass?	45	93.8	3	6.2
	2. Do the perfusionists interact with the surgical team to keep the blood flow rate in range?	42	87.5	6	12.5
	3. Are the changes in blood flow rate given to the surgical team?	36	75	6	12.5
	4. Is the speed of blood flow determined due to the assessment of anesthesia level, arterial blood pressure, cerebraloximetry, acid-base balance, lactate rate, the provided and consumed oxygen rate, systemic vascular strength, temperature, and venous blood saturation?	41	85.4	6	14.6
Gas exchange	1. Is the gas exchange in the patients during the cardiopulmonary bypass determined based on the appropriate instructions (patients' personal characteristics - type and the instruction to use oxygenator, – blood flow rate, temperature, metabolic needs of patients)?	43	89.6	5	10.4
	2. Is the equipment used to measure the gas exchange calibrated based on the manufacturer's instructions?	38	79.2	10	20.8
	3. Are the blood gases tests performed and recorded based on the instructions?	42	87.5	6	12.5
	4. Is it possible to perform the arterial blood gases tests in the operating room at any time?	41	85.4	7	14.6
	5. For the appropriate gas exchange, is the amount of the received and consumed oxygen calculated?	7	14.6	41	85.4
Blood transfusion and blood products	1. Do the perfusionists take the appropriate measures to prevent the hemodilution and blood transfusion and unnecessary blood products?	48	100	0	0
	2. In order to reduce the used prime volume, do the perfusionists attempt to decrease the length of the bypass?	46	95.8	2	4.2
	3. Before starting the cardiopulmonary bypass, do the perfusionists calculate the hematocrit and hemoglobin levels in patients after hemodilution and deliver the information to the surgical team?	35	72.9	13	27.1
	4. Is the clinical guide available for the blood transfusion and blood products during the cardiopulmonary bypass?	41	85.4	7	14.6
	5. In order to prevent the bleeding, is it possible to do the coagulation tests including INR, PTT, PT, Thrombin time, Platelet, and Platelet Function Analysis in the operating room?	41	85.4	7	14.6
Anticoagulants	1. Are the anticoagulants (heparin or alternative medicines in cases where the use of heparin is not appropriate) and the acceptable range of ACT determined based on the interaction between perfusionists and the physician in charge of the operation?	45	93.8	3	6.2
	2. Regarding the coagulation situation of patients before, during, and after surgery, do the perfusionists are in close interaction with the surgical team?	47	97.9	1	2.1
	3. Is the proper amount of ACT determined by the surgical team?	46	95.8	2	4.2
	4. Is the initial dose of heparin determined based on the proposed methods (weight, the prescribed dose response chart, blood volume, body mass index)?	48	100	0	0
	5. Is the anticoagulants monitoring performed based on ACT measurements?	48	100	0	0
	6. Are the other coagulation tests performed rather than ACT?	44	91.7	4	8.3
Perfusion report record	1. Is the perfusion report sheet recorded and maintained as one of the medical documents in patients' medical cases?	46	95.8	2	4.2
	2. Are the demographic information of the patient and risk factors recorded in the perfusion report sheet?	42	87.5	6	12.5
	3. Are the information related to the perfusion method, equipment and tools, and information of the perfusionists recorded accurately in the perfusion report sheet?	40	83.3	8	16.7
	4. Are the physiological variables in the specified times recorded based on the health center instructions in the perfusion repost sheet?	38	79.2	10	20.8
	5. Are the results of blood gases tests and coagulation tests recorded in the perfusion report sheet?	46	95.2	2	4.2
	6. Is the perfusion report sheet signed by the perfusionist in charge and alternative perfusionists?	40	83.3	2	4.2
	7. Are the oral instructions of perfusion recorded in the report sheet?	13	27.1	35	72.9
	8. Is report sheet signed by the physician in charge of perfusion?	2	4.2	46	95.8
	9. Is raw data (blood flow and pressure, temperature) recorded and made available in the electronic database for a specified period based on the health center instructions?	3	6.3	45	93.8

## Discussion and Conclusion

Regarding the significance attached to the blood pressure in the study centers, the blood pressure situation was relatively favorable for the standards of the questions 1, 2 and unfavorable for the standards of the questions 3, 4. For the questions 3 and 4, it could be stated that it was caused by the poor relationship between the perfusionist and the surgical team. There was a significant difference in the appropriate management of physiological variables during the bypass. The patients with lower risk tolerated the average arterial blood pressure 50–60 mm Hg without the complications, while there was low information about the blood pressure above 70 mm Hg in the patients with high risk,^6^ and finally the standards proposed necessitated that the blood pressure rate during the operation should be determined earlier through interaction with the surgical team and there should be an interaction during the operation between the surgical team to maintain the pressure healthy. The blood pressure changes should be recorded in the perfusion sheet and the measure be taken to prevent the blood pressure changes. The records should be given to the surgical team.^7^

Regarding the standard of blood flow rate, the situation of the centers on average on all the questions related to this standard was favorable. The standard proposed in this regard suggests that the target blood flow rate should be determined before the start of the bypass and be interacted with the surgical team in order to maintain the blood flow rate during the bypass. The blood flow speed was determined during the bypass through the assessments of the anesthesia level, arterial blood pressure, cerebraloximetry, acid-base balance, lactate rate, the provided and consumed oxygen rate, systemic vascular strength, temperature and venous blood saturation. The blood flow rate changes should be reported to the surgical team.^7^ There are two types of flow for creating blood pressure: pulsatile and nonpulsatile. Despite the controversies on the nonpulsatile flow effects on microcirculation, use of this type of flow is widely accepted in patients undergoing the cardiopulmonary bypass.^8,9^

Regarding the gas exchange standards, the situation of the centers was favorable for the standards of the questions 1-4 and unfavorable for the question 5. Considering the calibrated equipment used to measure the gas exchange, it could be stated that most of the study centers had rules for the equipment periodic services and it might be the reason for high scores in this section. For gas exchange during the bypass, usually the membrane oxygenators and the centrifugal and roller pumps were used. According to the proposed standards, it is necessary to be sure of the patient’s coagulation status before starting the bypass.^10^ According to the proposed standards by AmSECT, the gas exchange of the patients during the cardiopulmonary bypass should be determined based on the appropriate instructions (patients’ personal characteristics - type and the instruction to use oxygenator – blood flow rate, temperature, metabolic needs of patients) and the equipment used to measure the gas exchange calibrated based on the manufacturer’s instructions. The possibility of the tests related to the arterial blood gases should be provided in the operating room and recorded based on the appropriate instructions.^7^

Regarding the blood transfusion and blood products standard, on average, the centers were in appropriate situation. In the United States, almost 15×10^6^ red blood cells are used annually for the surgical procedures. Postoperative bleeding and clotting disorders, especially in patients who undergo the cardiopulmonary bypasses are considered as the major problems after cardiovascular surgery.^11^ Around 4%-10% of patients experience a noticeable loss of blood after the cardiovascular surgery and more than 25% of blood products used in the world are used for these patients.^12,13^ Despite the intense desire to maintain the blood and minimize the blood consumption, the number of annual consumption of blood is increasing.^14^ Blood donor is a rare resource and associated with the increased costs of healthcare and considerable risk for the patients.^15^ Dealing with the problem of bleeding and blood transfusion is important for at least two reasons: first, blood and blood products are limited and resources are expensive, and second, blood transfusion and bleeding are associated with increased mortality rates.^16,17^ According to the standards proposed by AmSECT, the perfusionists should use the measures to prevent the hemodilution and avoid the blood transfusion and unnecessary blood products. The length of bypass circuit should be shortened and a clinical guide should be developed for the blood transfusion and blood products.^7^

The study centers gave positive answers to all the questions related to the anticoagulants during the bypass which represented the appropriate situation, and it could be caused by the guideline and deplorable situation of the risks caused by the non-observation of the standards. Coagulation and inflammatory paths were stimulated through the blood contact with the cardiopulmonary bypass circuit and surgical wound. Activated clotting time (ACT) is a standardized coagulation test.^18^ Dose of heparin used to prevent blood clots during the cardiopulmonary bypass (CPB) 300-400 U/kg along with additional doses to achieve and maintain ACT is higher than 480s. However, the response of people to a fixed dose of heparin is different. High doses of heparin may be more successful in inhibiting thrombin; thus, they maintain the coagulation factors during the CPB.^19,20^

Regarding the perfusion record, the situation of the centers was favorable for the standards of questions 1-6 and unfavorable for the questions 7-9. Since the perfusion record sheet was sent to the centers by the Iranian Society of Extracorporeal Technology, in most of the cases it held the required standards but the sections related to the centers was usually unfavorable. Perfusion record is a legal record of the cardiopulmonary bypass procedure which should be perfectly legible and accurately documented. Traditionally, perfusion record was done manually in which perfusionists often documented the parameters and specific events every 5 to 10 minutes or recorded it when changes in the parameters of monitor occurred or a certain case happened (e.g. administrating medications and verbal commands). The handwritten perfusion record is the most common method to record the events during the cardiopulmonary bypass.^21^ This manual method of data collection of the procedure can be recorded as medical records; however, these records are often inadequate. Manual record often does not provide a complete picture of events during the bypass period and has a number of shortcomings. These shortcomings include missing information during critical events that put the perfusionists under the pressure, writing biased information, transcription error, and subjectivity of observation.^22,23^ In the hospitals, the information collection and recording might be used to improve the choice, surgical techniques, and the caring process of the patients undergoing cardiacsurgery.^24^

According to the results about the standard observation in perfusion performance in Iran, it could be concluded that although Iran is in favorable situation on observing the standard of anticoagulants, the blood transfusion and blood products, and the blood flow rate, the mentioned centers are not in appropriate situation on observing the standards of developing the institute-based protocols and equipment control using the checklists. The issue represents the necessity of authorities’ attention to the validation programs and development of the caring standards on one hand and the continuous assessment of using these standards on the other hand. Therefore, such studies are important for the health authorities to provide the caring favorable services and to conduct continuous assessment of these services.

## Limitations

Due to the self-responsiveness of people to the likelihood of random accountability to questionnaire and concealing the reality, only centers participated in this study which had a complete satisfaction to solve this problem.

## Acknowledgements

This study was part of a master thesis which was granted by Iran University of Medical Sciences. The authors would like to thank cardiac surgery teams in Shahid Rajaie, Cardiovascular, Medical and Research Center, Iranian Society of Extracorporeal Circulation Technology, and the perfusionists who participated to complete the questionnaires.

## Ethical approval

The study was approved by the Ethics Committee of Iran University of Medical sciences.

## Competing interests

Authors declare no conflict of interests in this study.
